# Sentinel lymph node excision with or without preoperative hybrid single-photon emission computed tomography/computed tomography (SPECT/CT) in melanoma: study protocol for a multicentric randomized controlled trial

**DOI:** 10.1186/s13063-019-3197-7

**Published:** 2019-02-04

**Authors:** Ingo Stoffels, Ken Herrmann, Jan Rekowski, Philipp Jansen, Dirk Schadendorf, Andreas Stang, Joachim Klode

**Affiliations:** 1 Department of Dermatology, Venerology and Allergology, University-Hospital Essen, University of Duisburg-Essen, 45122 Essen, Germany; 2 West German Cancer Center, University Duisburg-Essen, 45122 Essen, Germany; 3 German Consortium for Translational Cancer Research, Partner Site University Hospital Essen, Essen, Germany; 4 Department of Nuclear Medicine, University of Essen-Duisburg, 45122 Essen, Germany; 5 Center of Clinical Epidemiology, Institute of Medical Informatics, Biometry and Epidemiology (IMIBE), University Hospital Essen, 45122 Essen, Germany

**Keywords:** Malignant melanoma, Sentinel lymph node excision, SPECT/CT

## Abstract

**Background:**

Melanoma has become a growing interdisciplinary problem in public health worldwide. According to the World Health Organization, the incidence of melanoma is increasing faster than any other cancer in the world. Because melanoma metastasizes early into the regional lymph nodes, sentinel lymph node excision (SLNE) is included in the current American Joint Committee of Cancer guidelines. However SLNE of melanoma has a high false-negative rate of up to 44%.

**Methods:**

The gold standard for detection and extirpation of the sentinel lymph node is preoperative lymphoscintigraphy. SPECT/CT provides complementary information: the advantages include accurate anatomical localization, identification of false positives, reduction in the number of false negatives, and alteration of the surgical approach. Therefore, sentinel lymph node-SPECT/CT provides valuable information before sentinel lymph node excision and advocates its use in melanoma. We present a multicenter, unblinded superiority randomized controlled trial to compare SPECT/CT-aided SLNE versus standard SLNE in melanoma patients.

**Discussion:**

The primary efficacy endpoint is distant metastasis-free survival. Secondary endpoints comprise overall survival, disease-free survival, rate of local relapses within the follow-up period (false-negative rate of sentinel lymph node), number of positive sentinel lymph nodes (sensitivity, false-positive rate), complication rate, quality of life, quality-adjusted life years, inpatient days, and overall costs during hospital stays.

**Trial registration:**

ClinicalTrials.gov, NCT03683550. Registered on 20 September 2018.

**Electronic supplementary material:**

The online version of this article (10.1186/s13063-019-3197-7) contains supplementary material, which is available to authorized users.

## Background

Melanoma has become a growing interdisciplinary problem in public health worldwide. According to the World Health Organization, the incidence of melanoma is increasing faster than any other cancer in the world. Melanoma is the third most common cancer in Australia and the fifth in the USA. The American Cancer Society estimated that about 70,230 new melanomas were diagnosed in the USA during 2011 [1], resulting in about 8790 deaths [2]. However, melanoma accounts for < 5% of skin cancer cases, yet it causes > 75% of skin cancer deaths and thus poses a significant health issue and economic burden [3].

Because melanoma, depending on tumor depth, metastasizes early into regional lymph nodes [4, 5], sentinel lymph node excision (SLNE) is probably the most important diagnostic and potentially therapeutic procedure for melanoma patients [6, 7]. Recommendations for the use of SLNE for primary melanoma are therefore included in the current American Joint Committee of Cancer guidelines. Critics argue that the routinely performed SLNE is a cost-intensive surgical intervention with potential morbidity that does not offer patients any advantage in overall survival [8–11]. Moreover, the SLNE of melanoma has a very high false-negative rate of up to 44% [12, 13]. The current gold standard for detection and targeted extirpation of the sentinel lymph node (SLN) is preoperative lymphoscintigraphy. Single-photon emission computed tomography (SPECT)/CT provides complementary functional and anatomical information and has been shown to be superior to planar imaging in a number of indications [14]. The advantages include more accurate anatomical localization, identification of false positives (due to contamination or spillover from the injection site), reduction in the number of false negatives (visualization of nodes not seen on planar imaging), and alteration of the surgical approach. We thus believe that sentinel lymph node SPECT/CT can provide valuable information before SLN biopsy and advocate its use in a range of tumors such as truncal and head and neck melanomas. Due to the lack of prospective multicenter randomized trials, many clinicians are uncertain in regard to preoperative SPECT/CT imaging for SLNE in melanoma patients, especially regarding distant metastasis-free survival, overall survival, and costs.

Clinical experience and small non-randomized clinical trials reported on the advantage of preoperative imaging with SPECT/CT [15, 16].

In our own published single-center analysis on this topic in the SPECT/CT cohort, more SLN were detected than in the standard cohort (2.40 vs 1.87; *p* < 0.001). The number of positive SLN per patient was significantly higher in the SPECT/CT cohort (0.34 vs 0.21; *p* = 0.038). The local relapse rate in the SPECT/CT cohort was lower than in the standard cohort (6.8% vs 23.8%, *p* = 0.021), which prolonged disease-free survival (*p* = 0.019) [14]. This study confirms the improved identification of SLN in overweight and obese melanoma patients [17] as well as those with tumors in the head and neck area [18, 19]. According to the observations of Morton et al., SLNE leads to an improvement of life years and quality-adjusted life years [7]. Another recent study from our group on SLNE in the head and neck region showed that SPECT/CT resulted in superior aesthetic results (because of smaller incisions) and reduced operating time. It was also shown that with the use of SPECT/CT, it was feasible to perform SLN excision under local anesthesia, resulting in a tenfold reduction in operating cost (€32.65 with local anesthesia vs €334.57 with general anesthesia; *p* < 0.0001) [20].

As we demonstrated, SPECT/CT improves the detection rate of SLN compared with the standard procedure [14]. Moreover, we could show in a cost-effectiveness analysis that, by adding the described preoperative SLN imaging by SPECT/CT to the current practice of preoperative imaging, a reduction of hospital stay, realization of the surgical procedure in local anesthesia, and lower complication rates are possible. This leads to a clear reduction in costs [20]. The advantages of SPECT/CT in melanoma include a higher overall rate of SLN detection, the ability to detect SLNs in difficult to interpret planar studies, better detection of SLNs near an injection site and better anatomical localization.

These advantages can result in a change in patient management by altering the surgical approach. Overall, it appears that SPECT/CT is useful in patients with head and neck and truncal melanoma, in patients with difficult to interpret planar images, and in patients with either non-visualization of the SLN or unusual drainage patterns on planar images.

### The need for a trial

**Fig. 1:**
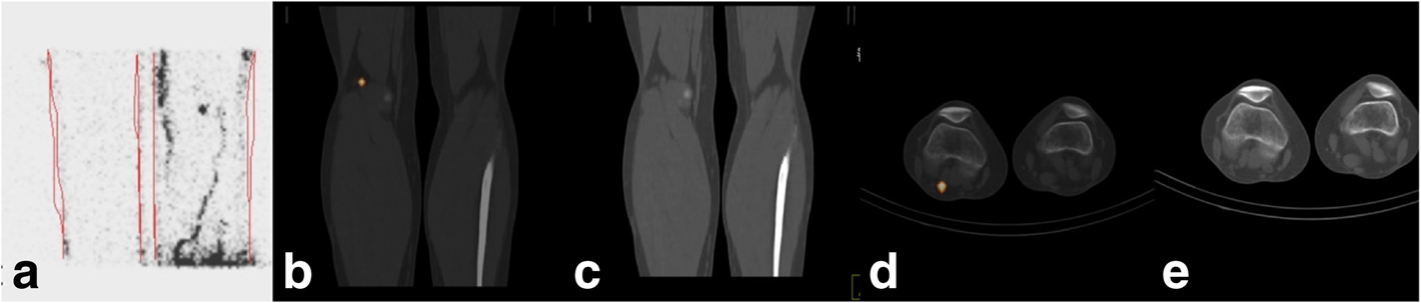
SPECT/CT. Patient with a malignant melanoma on the right foot. (**a**) Lymphoscintigraphy of the right popliteal region. (**b**) SPECT/CT in the sagital plane of the popliteal region with one SLN. (**c**) Low-dose CT in the sagittal plane of the popliteal region. (**d**) SPECT/CT in axial plane of the popliteal region with one SLN. (**e**) Low-dose CT in the axial plane of the popliteal region

The objective of the planned multicenter randomized prospective trial is to compare distant metastasis-free survival in patients with cutaneous melanoma between SLNE with versus without preoperative SPECT/CT imaging and metastatic node detection. The first objective of any additional imaging modality is to improve the detection rate of SLN metastasis and by this to prolong distant metastasis-free survival. The current gold standard for SLNE is planar preoperative lymphoscintigraphy. The use of an additional SPECT/CT technique offers the physician the preoperative possibility of determining the exact location and visualization of the SLN, especially if the tracer signal is too weak for detection by the handheld probe alone or the SLN is in the immediate vicinity of the remaining tracer depot (Fig. 1). However, there are no adequate randomized prospective trials available that would justify the use of preoperative SPECT/CT imaging by modern evidence-based treatment and patient safety standards. Due to the low level of evidence, the guidelines were not amended yet. Therefore, the current gold standard is the preoperative lymphoscintigraphy. The proposed trial would help to establish evidence-based recommendations for preoperative imaging in SLNE for melanoma patients.

## Methods/Design

### Overall study design and plan

This is a randomized, open-label, multi-center, superiority, two parallel arms trial comparing SLNE with or without preoperative hybrid SPECT/CT in patients with malignant melanoma.

**Fig. 2:**
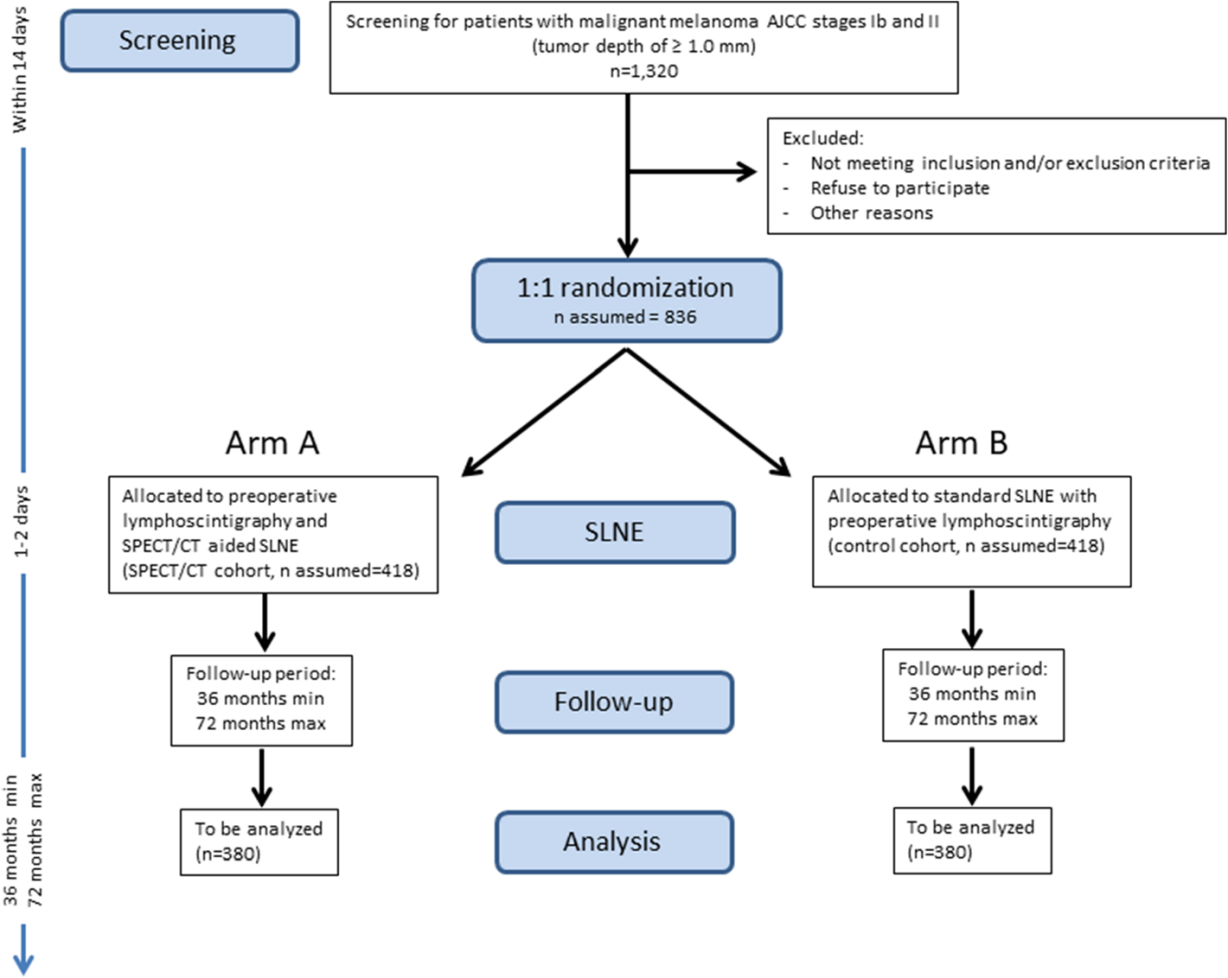
SNEPS Trial *flow chart*. FU follow-up, OP operation, SLNE sentinel lymph node excision, SPECT/CT single-photon emission computed tomography/computed tomography

The flow of the study is presented in Fig. 2. A SPIRIT Checklist [16] is included as Additional file  1.

**Fig. 3:**
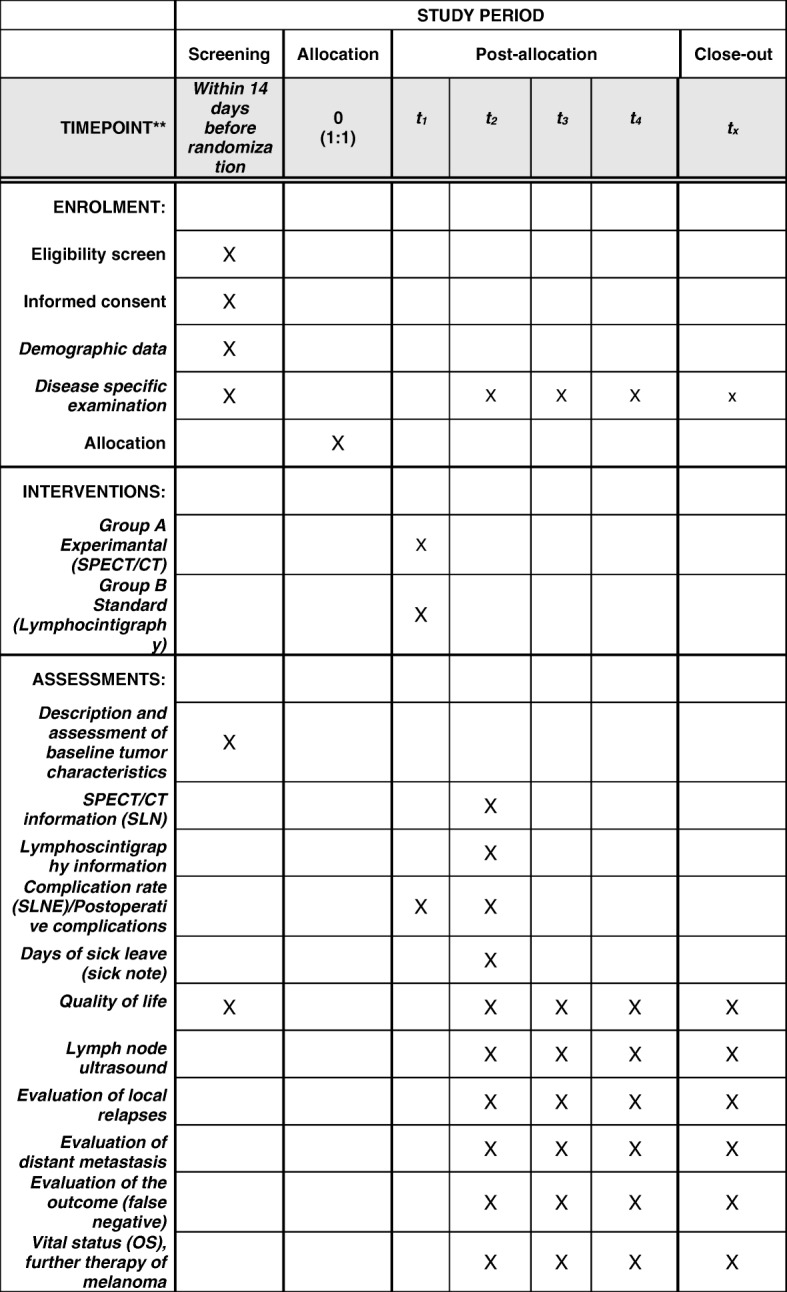
SPIRIT figure: schedule of enrolment, interventions, and assessments

**Fig. 4:**
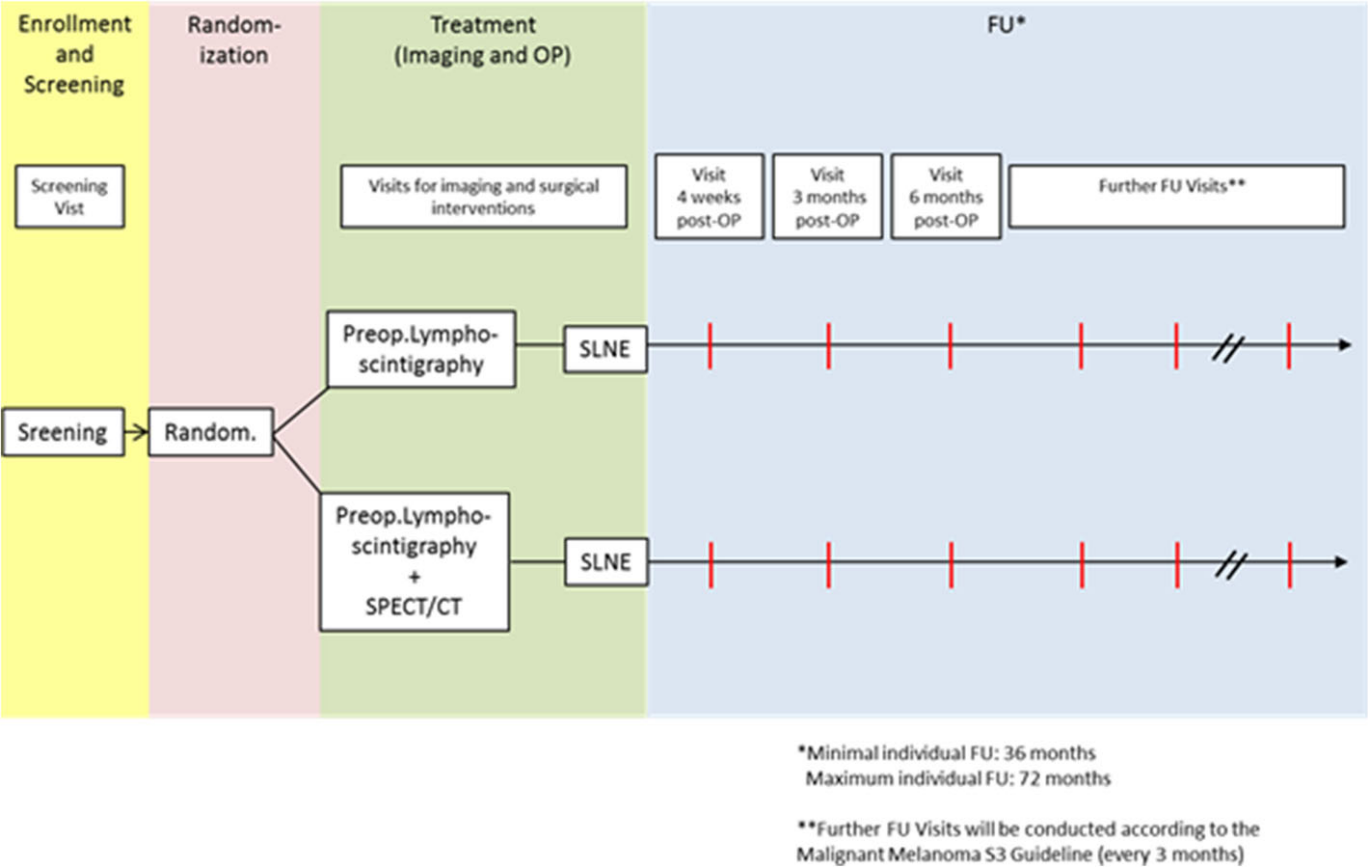
SNEPS visit scheme

### Randomization

Patients with malignant melanoma are allocated by 1:1 randomization to be treated with SLNE with or without preoperative hybrid SPECT/CT.

### Blinding

Not applicable as this is an open-label study.

### Study population and justification of choice of gender

After initiation of the sites, patients will be consecutively screened. The selection of patients occurs through the investigator according to the inclusion and exclusion criteria after informing the patient written and orally about the study and after the patient has signed the informed consent. There is no preferred enrolment of men or women within this study. However, pregnant or breast-feeding women are excluded from participation.

#### Inclusion criteria

Patients may be included in the study only if they meet all the following criteria:

- patients with malignant melanoma in American Joint Committee of Cancer stages Ib / II;
- tumor depth of ≥ 1.0 mm;
- age 18–75 years;
- have a primary melanoma that is cutaneous (including head, neck, trunk, extremity, scalp, palm, sole, subungual skin tissues);
- Eastern Cooperative Oncology Group (ECOG) performance status 0–1;
- life expectancy of at least five years from the time of diagnosis, not considering the melanoma in question, as determined by the principal investigator;
- willing to return to the trial center for follow-up examinations and procedures as outlined in the protocol;
- randomization must be completed no more than 120 days following the diagnostic biopsy of the primary melanoma;
- negative pregnancy test for female and effective contraception for both male and female participants if the risk of conception exists;
- signed written informed consent before the performance of any trial-specific procedure.

#### Exclusion criteria

Patients will be excluded from the study for any of the following reasons:

- history of previous or concurrent (i.e. second primary) invasive melanoma;
- primary melanoma of the eye, mucous membranes, or internal viscera;
- any additional solid tumor or hematologic malignancy during the past five years except skin lesions of squamous cell carcinoma, basal cell carcinoma, or uterine cervical cancer;
- skin grafts, tissue transfers, or flaps that have the potential to alter the lymphatic drainage pattern from the primary melanoma to a lymph node basin;
- hypersensitivity to the active substance(s), to any of the excipients or to any of the components of the labeled radiopharmaceutical;
- extensive previous surgery in the region of the primary tumor site or complete lymph node dissection or sentinel lymphadectenomy (before evaluation of the current melanoma) that may have altered the lymphatic drainage pattern from the primary cutaneous melanoma to a potential lymph node basin;
- organic brain syndrome or significant impairment of basal cognitive function or any psychiatric disorder that might preclude participation in the full protocol or be exacerbated by therapy (e.g. severe depression);
- pregnancy (absence to be confirmed by ß-HCG test) or lactation period;
- medical or psychological conditions that would not permit the individual to complete the study or sign informed consent;
- known alcohol or drug abuse;
- participation in another clinical study within the 30 days before registration;
- significant disease which, in the investigator’s opinion, would exclude the patient from the study;
- legal incapacity or limited legal capacity.

### Study treatment

#### Dose, mode, and scheme of intervention

Lymphoscintigraphy will be performed with either 16 MBq or 80 MBq of Technetium-99 m-nanocolloid (Nanocoll, GE Healthcare Buchler GmbH & Co. KG, Braunschweig) depending on the schedule of the surgical procedure: same day versus following day. The colloid will be injected in a total volume of 0.4 mL in four intradermal deposits of 0.1 mL each, which are located at the borders of the primary tumor site, or, if the primary tumor has been removed, will be located on both sides of the excisional scar. Dynamic images of the corresponding anatomical region and their adjacent lymphatic basins will be acquired at 30 s per frame for 5 min. With a total of 10 frames. Afterwards, anterior, lateral, and oblique projections were acquired for 5 min each, using a dual-detector gamma camera with a mounted two row multidetector CT scanner: SPECT = 128 × 128 matrix, 128 frames, 25 s/frame, OSEM algorithm with eight iterations and four subsets, correction for attenuation and scatter; CT = 130 kV, 17 mAs, 5 mm slices, image reconstruction in a medium smooth kernel.

The reconstructed data will be displayed as sagittal, coronal, and axial slices. Inherent image fusions will be generated from the co-registered SPECT and low-dose CT images using a software. Minor misregistrations will be corrected manually. Delayed planar images will be acquired 2 h after colloid injection, followed by SPECT/CT. If no SLN can be visualized in these images, another set of planar images will be acquired 2 h later.

Radioactive dosing is according to the “Verfahrensanweisung für die nuklearmedizinische Wächter-Lymphknoten – Diagnostik” of the “Deutsche Gesellschaft für Nuklearmedizin” and radiation protection. The resulting effective dose for the patient is small (< 3 mSv for SPECT/CT). Incidental findings of clinical relevance are reported to the treating physician. There are no equivalent diagnostic techniques for SLN labelling. The current gold standard for detection and targeted extirpation of the SLN is preoperative lymphoscintigraphy.

There will be no additional treatment. SLNE will be performed as a standard procedure according to the guidelines of the Deutsche Dermatologische Gesellschaft (DDG, German Association of Dermatology). The procedure will be offered to patients with malignant melanoma in AJCC stages Ib and II. The surgeons have experience in performing > 100 SLNE per year. Subsequent SLNE will be performed either under tumescent local anesthesia or general anesthesia. Preparation and subsequent excision of all radioactive marked lymph nodes will be carried out via a preoperatively marked incision. Surgery will be terminated as soon as no further radioactive foci could be traced in the surgical field. For all other examinations to be performed during the study refer to Fig. 3.

### Treatment plan

The visit scheme of the SNEPS trial is depicted in Fig. 4. Lymphoscintigraphy (and SPECT/CT in Arm A) for evaluation of sentinel nodes in combination with surgical intervention can be performed in one day or over two days. In case of a two-day protocol, the radiation of the radioactive substance injected on day 1 is sufficient for surgery on day 2.

Therefore, one or two treatment visits (imaging and surgical intervention) are planned per patient. Patients will be followed up for a minimum of 36 months (follow-up visits and examinations to be performed every three months are specified).

### Statistical methods

The primary analysis of efficacy and superiority of SPECT/CT-aided SLNE versus standard SLNE regarding distant metastasis-free survival will be tested confirmatorily on the intent-to-treat analysis set with a significance level of 5% by a two-sided test [21] of the regression coefficient for the treatment variable from a Cox proportional hazards model [22, 23]. This test coincides with the log-rank test. It is more suitable than the weighted relatives of the log-rank test because treatment achievements are expected to be observed at later points in time. The analysis with the Cox model will adjust for the factors of the randomization, that is site, sex, tumor depth (< 4.0 mm or ≥ 4.0 mm) and tumor location (head and neck region, yes or no). Due to the nature of the primary outcome variable, there will not be any missing data for it. No imputation of missing data has to be performed for the primary analysis. First, the realization of a time-to-event variable as distant metastasis-free survival is either an event or a censored observation, and thus, never missing. Second, missing values for the independent variables in the Cox proportional hazards model are neither possible as these variables are the factors of the randomization and the randomization cannot take place with one or more of its factors being missing.

Results will be displayed as adjusted Kaplan–Meier curves and reported as adjusted hazard ratios (HR) from the Cox proportional hazards model with corresponding 95% confidence intervals (CI). Sensitivity analysis for the primary endpoint will be performed using Kaplan–Meier curves and HRs not adjusting for the factors of the stratified randomization. Furthermore, a per-protocol analysis, adjusted and unadjusted for the stratification variables, will be conducted. Another sensitivity analysis will account for the fact that study patients may withdraw from this trial between randomization and SLNE as they may not be satisfied with the study group they were assigned to. Thus, we will create a worst case scenario treating all study patients who withdraw from the trial between randomization and SLNE as if they had the primary outcome event at randomization. Adjusted and unadjusted analyses will be repeated under this worst case scenario.

Because treatment achievements are expected to be observed relatively late in time, no interim analysis will be performed. Subgroup analyses will be carried out in the levels of the randomization factors.

Standard safety analyses will be done for all serious adverse events (SAEs) reported and documented during the trial period. Additionally, SAEs from the five most common system organ classes of the Medical Dictionary for Regulatory Activities (MedDRA®) will be pooled by system organ classes and analyzed as time to SAEs occurrence with the Anderson–Gill model [22] that can account for recurring events with cause-specific hazards in the case of competing risks. Furthermore, as recommended recently [24], the mean frequency function [25] will be used to allow the comparison in terms of absolute event probabilities.

All secondary analyses will be done exploratively, i.e. without adjustment for multiplicity. Standard statistics appropriate for the given level of measurement of the respective outcome will be applied and for evaluating the diagnostic performance of SPECT/CT aided SLNE (e.g. sensitivity, false-negative rate). For secondary analyses, imputation of time-to-event variables or factors used in the Cox proportional hazards model is not necessary due to the same reasons as for the primary analysis. For the remaining endpoints, where missing data are possible, multiple imputation methods are used with all factors of the randomization being included in the imputation model. In the sensitivity analysis, missing outcome data are replaced using a worst case scenario.

### Sample size

Sample size calculations have been carried out with PASS 13 Power Analysis and Sample Size Software [PASS 13, 2014] using the formula of Schoenfeld [26]. In a non-randomized clinical trial at the university hospital Essen with 402 melanoma patients, we observed a five-year distant metastasis-free survival cumulative survival probability of 67% determined using the Kaplan–Meier product limit estimator for patients after standard SLNE (n = 254, 46% with T2 and 31% with T3) which is in line with Morton et al. [27]. In the SNEPS trial, we intend to include patients of stages I and II but with an expected classification of mainly stage II or III after postoperative histology. We therefore assume to observe a comparable treatment effect in the control group. For patients with SPECT/CT-aided SLNE (*n* = 148), we observed a five-year distant metastasis-free survival cumulative survival probability of 77% in the non-randomized clinical trial. This corresponds to a HR of 1.56 (95% CI 1.06–2.30). This HR seems relatively large at first sight, but given the high distant metastasis-free survival rates it is equivalent to a difference in five-year distant metastasis-free survival of 10%, which is rather small for such a late time point, but still clinically relevant and in the range of generally accepted effect sizes in oncology trials.

The sample size calculation is based on the intention to compare the distant metastasis-free survival times in both groups with the partial likelihood method of Cox [21] that tests whether the regression coefficient from a Cox proportional hazards model [28] is equal to zero and that coincides with the log-rank test [24]. Given the two-sided version of this test with a significance level of 5% and assuming a true HR of 1.56, 214 events have to be observed in total to achieve a power of 90%, 126 in the control group, and 88 in the experimental group. These results assume that the HR is constant throughout the study. For the anticipated proportion of patients having a distant metastasis during the study, we assume 33% in the control group and 23% in the SPECT/CT-aided group. Thus, 380 evaluable patients per group (760 in total) have to be recruited. To account for 10% dropouts or loss of information because of non-compliance to follow-up visits, a total of 836 patients will be randomized.

## Discussion

In this multi-center, unblinded superiority clinical trial SPECT/CT-aided SLNE versus standard SLNE in melanoma patients will be compared. The primary efficacy endpoint is distant metastasis-free survival. Secondary endpoints comprise overall survival, disease-free survival, rate of local relapses within the follow-up period (false-negative rate of SLN), number of positive SLN (sensitivity, false-positive rate), complication rate, quality of life, quality-adjusted life years, inpatient days, and overall costs during hospital stays.

Because melanoma, depending on tumor depth, metastasizes early into regional lymph nodes [4], SLNE is probably the most important diagnostic and potentially therapeutic procedure for melanoma patients. Recommendations for the use of SLNE for primary melanoma are therefore included in the current American Joint Committee of Cancer guidelines.

All study patients will at least receive the gold standard (lymphoscintigraphy). However, this procedure has resulted in some controversies in international discussions [29, 30]. Therefore, two diagnostic modalities will be compared. In the experimental study, one group of patients will additionally receive SPECT/CT. No additional risk is expected. From the experts of the German Association for Radiology, the radiologic diagnostic procedures in this study are evaluated as being according to medical standard so that no approval from the federal authority for radiation protection is required. Central ethical approval has been confirmed from Institutional Review Board of the University Hospital Essen (ref. approval no. 18–8288-BO) and we will not begin recruiting at other centers in the trial until local ethical approval has been obtained.

All investigators and the involved contract research organization (Alcedis GmbH, Gießen) ensure that the requirements of the World Medical Association Declaration of Helsinki are met and that the conduct of the trial is in accordance with current ICH guidelines for good clinical practice.

### Methods against bias

After initiation of the sites, all patients will be consecutively screened and all eligible patients willing to participate will be included in the trial. In order to achieve comparable groups regarding possibly relevant predictors and achieve balanced group sizes per site, patients will be allocated in a concealed manner by central web-based preoperative 1:1 stratified (minimization algorithm [Pocock and Simon] with biased coin principle) randomization with stratification factors site, sex, tumor depth (< 4.0 mm or ≥ 4.0 mm), and tumor location (head and neck region, yes or no). The primary analysis will control for the factors of the randomization. Each patient who is registered will be randomized and each randomized patient is part of the intent-to-treat analysis. It is not feasible to blind the patient or initial reader of the SPECT; however, physicians responsible for the follow-up and detection of distant met\astases will be blinded against the SPECT status by non-accessibility of the SPECT electronic case report form. In addition, the local interdisciplinary tumor board and an independent endpoint committee will review written reports of potential outcomes. The surgical technique will be identical in all participating sites and only operating physicians with a cumulative experience > 100 SLN surgical procedures will participate. The impact of a learning curve is thereby small. Performance bias will be avoided to a certain extent by certifying only experienced surgeons. All participating sites in Germany are certified by OncoZert and have a quality management system. All clinical investigators involved in this trial are experienced in performing SLN surgery, using preoperative SPECT/CT imaging and are trained in Good Clinical Practice. This allows for reduction of interrater variability to a minimum.

## Trial status

Protocol version 1.1 (19. September 2018). The trial began recruitment on 25 September 2018 and is currently ongoing.

## Additional file

Additional file 1: SPIRIT Checklist. The complete SPIRIT checklist regarding the SNEPS trial. (PDF 215 kb)
